# Experiences of using a supported digital intervention for cancer survivors in primary care: a qualitative process evaluation

**DOI:** 10.1007/s11764-023-01412-2

**Published:** 2023-07-05

**Authors:** Jazzine Smith, Rosie Essery, Lucy Yardley, Alison Richardson, Joanna Slodkowska-Barabasz, Cassandra Chavlet, Claire Foster, Eila Watson, Chloe Grimmett, Adam W. A. Geraghty, Paul Little, Geoffrey Sharman, Tamsin Burford, Roger Bacon, Lesley Turner, Katherine Bradbury

**Affiliations:** 1 School of Psychology, University of Southampton, Southampton, UK; 2 School of Psychological Science, University of Bristol, Bristol, UK; 3 School of Health Sciences, University of Southampton, Southampton, UK; 4 University Hospital Southampton, Southampton, UK; 5 Community and Public Health Faculty of Health and Life Sciences, Oxford Brookes University, Oxford, UK; 6 Primary Care Research, University of Southampton, Southampton, UK; 7 Patient and Public Involvement Team, Southampton, UK

**Keywords:** Process evaluation, Digital intervention, Primary care, Quality of life, Patient experience

## Abstract

**Background:**

Increasing healthy behaviours (e.g. physical activity) can improve cancer survivors’ quality of life. Renewed is a digital intervention developed to provide behaviour change advice with brief healthcare practitioner support. A three-arm randomised controlled trial (Renewed, Renewed with support or a control condition) suggested that prostate cancer survivors in the supported arm had slightly greater estimates of improvements in quality of life compared to other cancer survivors. This study explored participants’ experiences using Renewed to understand how it might have worked and why it might have provided greater benefit for prostate cancer survivors and those in the supported arm.

**Methods:**

Thirty-three semi-structured telephone interviews with cancer survivors’ (breast, colorectal, prostate) from the Renewed trial explored their experiences of using Renewed and their perceptions of the intervention. Data were analysed using inductive thematic analysis.

**Results:**

Some participants only used Renewed modestly but still made behaviour changes. Barriers to using Renewed included low perceived need, joining the study to advance scientific knowledge or ‘to give back’, or due to perceived availability of support in their existing social networks. Prostate cancer survivors reported less social support outside of Renewed compared to participants with other cancers.

**Conclusion:**

Renewed may support healthy behaviour changes among cancer survivors even with limited use. Interventions targetting individuals who lack social support may be beneficial.

**Implications for Cancer Survivors:**

Cancer survivors’ experiences may inform the development of digital interventions to better serve this population.

## Introduction

There are an estimated three million cancer survivors in the UK, projected to increase to 5.3 million by 2040 [1]. Those that complete primary treatment are at greater risk than the general population of developing several health-related problems during the transition from treatment to life after cancer [2]. These include anxiety, depression, fatigue and weight gain, contributing to reduced quality of life (QoL) [1]. Following this increase in the growing number of cancer survivors, there is rising demand on National Health Service (NHS) cancer services [3].

Digital interventions may help improve cancer survivors’ QoL through providing support with issues like psychological distress management, physical activity, healthy diet and weight management [4]. A digital intervention, “Renewed” [5, 8], was developed to target multiple health behaviours in order to improve QoL of cancer survivors. Renewed includes the option of brief health care professional (HCP) support with the aim of being implemented across NHS primary care services. The addition of HCP support alongside a digital intervention could be of perceived value for cancer survivors [9] and an important factor in increasing engagement [10, 11].

A randomised control trial (RCT) of Renewed compared improvements in quality of life among cancer survivors who were randomised to either (1) Renewed: web-only intervention, (2) Renewed with support: web-based intervention with additional guidance and (HCP) support or (3) Control: Generic advice and follow-up. Cancer survivors in the control arm were given a link to the NHS Live Well website which provides support for mental health, healthy eating, exercise, sleep, smoking and alcohol, sexual health and addiction [12]. Results showed that the impact appeared to be slightly greater for those with prostate cancer who were given access to human support compared to those with breast or colorectal cancer.

Whilst Renewed has already been the subject of evaluation through an RCT, when evaluating a complex intervention, it is important to also explore how the intervention worked, for whom and under what circumstances. Thus, combining process evaluations with RCTs can enable intervention developers and evaluators to develop a detailed understanding of how the intervention worked that can support stakeholders in interpreting effectiveness [13]. Qualitative process studies can explore how users interact with an intervention to produce effects and why users did or did not use the intervention as intended [14]. For example, our previous qualitative process study exploring HCPs experiences supporting those using Renewed found that an approach where the expertise is provided by the intervention and brief additional support provided by a healthcare professional is an acceptable way to overcome key barriers to supporting cancer survivors in primary care. Additionally, whilst most HCPs cope well with delivering non-directive support, a minority may need more support to feel confident implementing this [15]. Similarly, understanding how cancer survivors interact with digital interventions like Renewed and what may serve as potential barriers or facilitators could inform the design of future digital interventions for this group and others with long-term conditions and may also have implications for how cancer survivors can be best supported in primary care [14]. A process evaluation can also help to understand the reasons why certain groups appeared to benefit more than others [11, 14]. Following guidance for conducting process evaluations, interviews were conducted and analysed before knowledge of the RCT outcomes to avoid biased interpretation [13, 14]. The findings were then considered alongside the trial results when these became available later. Whilst data were collected before trial outcomes were known, analysis was not completed until final trial outcomes were known so that data could be used to examine trial findings. This allowed exploration for potential explanations of trial findings.

Therefore, this study aimed to explore how and why cancer survivors used Renewed as they did. Specifically, it aimed to explore (1) what factors may serve as potential barriers and facilitators to cancer survivors’ using Renewed and performing the recommended behaviours?; (2) why Renewed may have provided more benefit to prostate cancer survivors than other cancers?; (3) any perceived changes in participants’ quality of life and how these were experienced whilst engaging with Renewed.

## Methods

### Design

This qualitative process evaluation was nested within the Renewed RCT [5]. Cancer survivors were invited to participate by their GP surgeries. After online screening, they completed baseline measures via Renewed before being randomised to one of three conditions: (1) Renewed, (2) Renewed with support or (3) Control arm. The process evaluation employed semi-structured qualitative interviews to explore participants’ experiences of participating in the Renewed RCT and using Renewed (arms 1 and 2). The COnsolidated criteria for REporting Qualitative studies (COREQ) checklist [16] was used to guide reporting of this study.

### Intervention

Renewed is a web-based digital intervention designed to provide support for increasing multiple healthy behaviour changes among cancer survivors to increase their QoL. The Renewed intervention includes a website Renewed (arm 1) and optional brief support from a healthcare professional (arm 2). Renewed begins with an introductory session, ‘Core Content’, which contains information on the benefits of healthy behaviour changes and brief advice on how to make behaviour changes, with signposting to other resources. Users on active surveillance with prostate cancer receive additional information about active surveillance to provide reassurance, as this group can be anxious about monitoring being used in place of treatment [17]. Users are then given personalised suggestions of how the optional components within Renewed can help them based on answers to a quality of life measure (European Organization for Research and Treatment of Cancer measure (EORTC; [18]). There were four optional components in Renewed: Getting Active, Eat for Health, Healthy Paths [19] and POWeR [10, 20–23]. Cancer survivors randomised to the Renewed with support arm had the option of brief support sessions with a healthcare professional. Table 1 describes the Renewed intervention in more detail.

**Table 1:** Renewed intervention descripton

Components	Description
Core Content	Contains an introductory session which provides an overview of what to expect from Renewed. Renewed then provides tailored suggestions about which components of the programme would be most helpful for managing the particular symptoms each participant is experiencing, based on answers to a quality of life measure (their European Organization for Research and Treatment of Cancer score (EORTC; [18]) response. Links are given to additional resources not provided by Renewed (e.g. financial help, community support, return to work). Users undergoing surveillance for prostate cancer are provided with reassuring information about the safety and efficacy of active surveillance. After completing the Core Content, users are introduced to the homepage, from where they can access the other components of Renewed
Healthy Paths	Healthy Paths is designed to reduce stress and improve mental health well-being, through mindfulness-based and cognitive behavioural therapy (CBT) techniques. Cancer-specific modules provide techniques for dealing with fear of cancer recurrence and feelings of loss following cancer. Links to support the management of difficult feelings and emotions are provided
Getting Active	Getting Active is designed to encourage moderate physical activity through a range of interactive components and behavioural change techniques. An initial quiz is used to increase motivation for physical activity and address cancer-specific physical health concerns (i.e. fatigue, pain). Suggestions for increasing physical activity gently are given, and participants can then choose an activity option, such as exercising at home or walking, before being encouraged to set achievable personal goals. Goals are reviewed weekly, and tailored feedback is provided. Users were given the option to order a free step counter. Links to other physical activity resources are provided (e.g. benefits of physical activity, local activities near users)
Eat for Health	Eat for Health was designed to enhance knowledge of healthy eating and increase motivation to make changes to eating habits—a diet which is high in fruit and vegetables and low in fat, sugar, alcohol, and red/processed meats. Participants complete a short quiz to learn about the benefits of healthy eating. Common concerns about changing diet are addressed, and an easy to follow eating plan is presented which uses a traffic light system. Meal plans and healthy eating recipes are also available. Participants are encouraged to set healthy eating goals which can be reviewed and updated. Tailored feedback is provided. Eat for Health provides a goal setting and reviewing facility to enable self-monitoring of diet. Additional links to support healthy eating are provided (e.g. drinking alcohol, eating problems)
POWeR + (Positive Online Weight Management)	POWeR is a digital weight management intervention shown to be effective, described in full elsewhere [20]. Participants can choose between a low calorie/low carbohydrate eating plan and a walking/any other physical activity plan. POWeR provides physical activity support (e.g. walking or any other physical activity). Weight and goals are reviewed weekly, and tailored feedback is provided. Twenty-five sessions provide strategies to support weight loss (e.g. coping with cravings, relapse prevention)
Optional support sessions *(for those in the ‘Renewed Online with brief human support’ group)*	Ten-minute support sessions were offered at 2, 4 and 8 weeks after patients had begun the study via telephone or face-to-face. The role of the Supporter was to provide a listening ear to help patients decide which changes they might like to try, encourage patients to try out a change or keep going with changes. Supporters were asked not to give advice; rather, all advice would come from Renewed. Instead, they were asked to use the ‘CARE’ approach: congratulate, ask, reassure and encourage [24]. CARE aimed to facilitate an autonomous supportive relationship which promotes patient empowerment and aimed to achieve longer-term adherence to behaviour changes [24]

### Participants

Patients were eligible for the Renewed trial if they had finished treatment for breast, prostate or colorectal cancer within the last 10 years, or were on active surveillance with prostate cancer. Additional eligibility included self-reported reduced QoL (as defined by scores < 85 on EORTC QLQ-C30 [18]) and access to the Internet. Full inclusion criteria can be found in the RCT protocol [5].

Participants for the qualitative process study were sampled from two arms of the Renewed trial and invited to take part in telephone interviews. Purposive sampling was used to target a maximum variation across factors that might influence the intervention’s acceptability or effectiveness. These included age, gender, years since finishing treatment, education level, cancer type and level of Renewed usage. Usage was categorised into two groups: (1) those that only accessed the Core Content (low users) and (2) those that completed the Core Content and accessed at least one other component of Renewed (high users).

### Procedure

Patients were identified for interviews through the Renewed participant database and invited via email or phone calls. Following online informed consent, interviews were conducted via telephone between February and April 2019 by two trained qualitative interviewers (JS, JSB). Interviews ranged from 9 min to 1 h and 30 min, and the median interview length was 26 min. Whilst most interviews were close to the median time length, a couple interviews were longer than an hour due to participants’ tangential responses. One interview was 9 min due to the participant no longer wanting to continue the interview. Further interviews to capture any differences in patients’ experiences of using Renewed during COVID-19 were conducted by CC, who received training in qualitative interviews.

Semi-structured interview schedules were developed by a qualitative researcher (JS), who was not involved in the development of Renewed, and a health psychologist and experienced qualitative researcher (KB). Open-ended questions were used to allow participants to freely describe their experiences and views in their own way and to focus on whatever was most salient to them. Topics covered included: experiences of using the Renewed intervention, any behavioural changes made whilst being in the Renewed study, experiences of healthcare professional support received within Renewed and experience of using Renewed during the COVID-19 pandemic.

### Analysis

Individual interviews were audio‐recorded, transcribed verbatim and anonymised. Inductive thematic analysis was conducted using Braun and Clarke’s [25] 6-step process to develop themes related to patients’ experiences of using Renewed and being in the study. A charting framework was used to support comparisons across participant characteristics (e.g. cancer type and usage levels, and Renewed trial arms [26]. Identification and validation of developing themes were achieved through an iterative process of data analysis with frequent discussions between JS, KB and RE. Data were collected concurrently to data analysis, allowing sampling to be adapted to reflect analytic insights. Coding was performed using NVivo software (Version 12.0.0 [27]). Deviant cases were considered to ensure that minority views were not overlooked [28]. A coding manual was developed which was updated to reflect the ongoing analysis [29]. An audit trail and reflective log were completed to maintain rigour during analysis.

## Results

### Participant characteristics

Thirty-six participants were interviewed. Data from three participants were excluded from the analysis as they could not remember using Renewed and/or being in the study. Thirty-three participants were included in the analysis; 16 were in the *Renewed with support arm* and 17 were in the *Renewed arm*. The demographic and clinical characteristics of the included participants are reported in Table 2.

**Table 2:** Participant characteristics

Baseline characteristics	
Age (years)	
Mean (S.D)	62.8 (10.20)
Range	36–82
Baseline EORTC QLQ-C30 score	
Mean (S.D)	73.5 (11.3)
Range	39.4–84.1
Cancer group	
Colorectal	10/33 (30.3%)
Breast	14/33 (42.4%)
Prostate	7/33 (21.2%)
Prostate active surveillance	2/33 (6.1%)
Renewed RCT group	
Renewed	17/33 (51.5%)
Renewed with support	16/33 (48.5%)
Gender	
Male	14/33 (42.4%)
Female	19/33 (57.6%)
Ethnicity	
White	33/33 (100%)
Time since last cancer treatment (years)	
Mean (S.D)	3 (2.9)
Range	0–9
Age when left education (years)	
Mean (S.D)	18 (3.5)
Renewed usage	
Accessed up to the Core Content	19/33 (58%)
Accessed the optional content	14/33 (42%)
Support sessions (for those in the ‘Renewed with support arm)	
Accessed support	9/16 (56%)
Chose not to access support	7/16 (44%)

### Themes

Four themes were developed: (1) Using Renewed to support behaviour change, (2) Patient’s perceived need for support from Renewed, (3) Barriers to using Renewed and performing behaviour changes and (4) Personal touch and added value of human support. The themes contribute to an understanding of perceived changes in quality of life and how these related to engagement with Renewed, why Renewed may have provided greater benefit for prostate cancer survivors and factors that may have served as potential barriers and facilitators to patients’ engagement with Renewed and the recommended behaviours. The analysis considered the role of participant characteristics (age, gender, the year participants finished education, years since end of treatment, cancer type and Renewed usage), in the accounts of their experiences, but analysis did not reveal any noticeable differences based on age, gender and the age participants finished education. The results include an illustration of different experiences within these themes relating to both cancer type, level of Renewed usage and years since finishing cancer treatment. Representative quotes are included to illustrate key points. Participants are referred to by their ID number, Renewed usage level, cancer type and Renewed RCT trial arm to provide contextual understanding.

### Using Renewed to support behaviour change

- Renewed supported autonomy with behaviour change

Many patients expressed that they liked being able to use Renewed at their own pace and in their own time and locations. They highlighted the benefit of choosing what to look at on Renewed and deciding which behaviours they wanted to perform. The majority of participants expressed that they found Renewed easy to access and use, having access from their own home, instead of having to travel to a GP. Also, the ability to go back and review information and activities made Renewed more accessible to many patients’ schedules and learning patterns.

You can take whatever you want…choose and change, you don’t have to keep to one plan. If you’ve got more confident you think ‘oh well, I’ve done this but, later on I can do a bit of this also’…it always reminds you also that if you don’t have time now you can go back on the home page, so it doesn’t put pressure on you. *(Participant 16, high user, 56 years old, female, breast cancer, Renewed arm).*

Patients described being able to use Renewed in a way that best suited their needs and goals. For example, within Renewed, feedback about which parts of Renewed an individual may find most helpful was given at the end of the Core Content, based upon patients’ answers to a quality of life measure which highlighted the symptoms patients were finding most bothersome. Patients sometimes reported this feedback helped them to make a decision about which behaviour changes to perform.

The programme [Core Content] suggested the websites [components] I might like to look at, like the POWeR one for losing weight ‘cause that, identified the things that I needed to work with. And I thought that was really good. So the things that I focussed on was the losing weight one. And the exercise basically. So I didn’t really look at anything more than that. *(Participant 15, high-user, 65 years old, female, breast cancer, Renewed arm).*

However, the majority of the time, participants already had an idea of which behaviour changes they wished to undertake, regardless of the feedback provided at the end of the Core Content. For example, one patient who had been recommended all components of Renewed, spoke of not choosing to use Healthy Paths because she knew what other behaviours she wanted to work on.

I never did the Healthy Paths one, because it wasn’t really a priority for me…I was more interested in the three that I have used, because it was focussing me on, you know, I want to keep my health up, and I want to keep fit. But weight’s a problem, and it [Renewed] focusses you, I think. *(Participant 20, high-user, female, colon cancer, Renewed arm).*

b) Engagement with Renewed related to ‘offline’ behaviour change

Patients were also able to choose to use Renewed as little or as much as suited them. As a result, there were differences in reported changes in behaviour based on patients usage level. It appeared that many patients who only accessed the Core Content reported no, or very few, changes in their behaviour.

Looked around it, but haven’t really taken up on any of the suggestions it makes. *(Participant 22, low-user, male, colon cancer, Renewed arm)*

On the other hand, a few patients who only accessed the Core Content expressed that whilst they may not have used Renewed much, it was enough to begin making behaviour changes.

I followed some of the diet advice. And taking yourself off out for a walk and things like that, which I did try. Just to make my lifestyle a bit healthier. *(Participant 8, low-user, female, breast cancer, Renewed arm)*

Similarly, many high users appeared to use Renewed to begin making behaviour changes. They would sometimes stop using Renewed once they had accessed what they perceived as sufficient information to implement the changes, often with support from their own ‘offline’ tools and resources (i.e. Fitbit, calendars).

Trying to get my weight down, that sort of thing. I found that all that very useful and I made up the little calendar thing, but once you referred to these suggestions on the site, I didn’t really feel a great deal of need to go back to them, because I put what I could into action, and did it. *(Participant 6, high-user, male, prostate cancer, Renewed with support arm).*

### Patient’s perceived need for support from Renewed

Patient motivation to use Renewed often appeared to be determined by whether they perceived a need for the type of support Renewed offered. Several factors seemed to contribute to patients’ perceptions of need for this type of support. For example, some patients put emphasis on the importance of learning something new from Renewed. A few high-users reported that the content of Renewed was novel.

One of the sections [Healthy Paths] put you onto the BBC one, which I’ve used before. But it put me down the clean eating one, which I hadn’t really considered…So when you look at the recipes, it reminds you to have snacks like walnuts or something. And they’re things that you don’t necessarily think about…So sometimes if you have it written out for you, which it was in this case, you just think, I might try that, or give that a go, or that’s a good idea. *(Participant 20, high-user, female, colon cancer, Renewed arm)*

However, many low users felt that it was too basic and did not teach them anything they did not already know or were already doing. In these instances, patients would often not continue to use Renewed beyond the Core Content where they would have been exposed to the more detailed and novel content that was contained in the optional content.

I would say I’m actually quite well informed but for a lot of people that aren’t, it’s very useful. I thought I knew enough about my dietary stuff. *(Participant 27, low-user, female, breast cancer, Renewed arm).*

A few patients did not find Renewed suitable for them, as they believed they already lived a healthy lifestyle.

I go cycling for exercise. And I’ve kept that up as much as I am possible…I thought, ‘yes, been there, done that. *(Participant 22, low-user, male, colon cancer, Renewed arm)*

Two patients spoke of not being motivated to use Renewed because it had been a whilst since finishing their treatment, and they felt as if they were no longer in need of this sort of support.

And perhaps five years ago, when I was five years in and just coming out of the treatment and starting anew, without help, then I was on my own, you know, after the five years of treatment when you’re seeing someone all the time, it would’ve been absolutely perfect. *(Participant 28, low-user, female, breast cancer, Renewed with support arm)*

A few individuals who did not engage much with Renewed appeared not to be especially motivated to use Renewed in order to make behaviour changes because they reported having existing resources which they could use to support them and preferred to use those.

I was already making my own changes with Lighter Life…So there was really nothing there that I could take up. *(Participant 32, low-user, male, prostate cancer, Renewed arm)*

In contrast, for a few others, a lack of social support in their lives motivated usage of Renewed. For example, deviant case analysis showed that one high user expressed that they used Renewed frequently because they did not get much support outside of Renewed. This user felt that aspects of Renewed such as progress monitoring, goal reviewing and email prompts gave a sense of support and community, which encouraged them to revisit Renewed.

I wasn’t really understood within my environment...when I told them [people within environment] that I had the cancer, they said no, it’s because I’ve put on weight. So I had to cope with them not accepting that I had the cancer… But the fact that actually I got that letter [Renewed study invitation] and people want to try and help me to get back into form, it’s really a help for me because I know that people actually do take an interest. *(Participant 16, high-user, female, breast cancer, Renewed arm)*

### Barriers to intervention use and behaviour change

Those with other health problems or with physical limitations to going outdoors experienced difficulty in actually performing the behaviours recommended by Renewed. Many patients often reported finding it difficult to perform some of the recommended behaviours alongside comorbidities, particularly those whose mobility was restricted or performance of a behaviour could cause immediate discomfort (e.g. trying to do exercise with an existing back problem). In one case, a patient who reported experiencing depression suggested that this interfered with his availability to use Renewed.

I’ve not really sort of liked touched on it [Renewed], because of my own mental health problems [depression] I’ve not really sort of like got into it, really. I’ve been sort of preoccupied. *(Participant 18, low-user, male, prostate cancer, Renewed with support arm)*

A few participants also reported adjusting to life changes (i.e. living with a stoma) after finishing treatment. These changes took priority over their motivation to make healthy behaviour changes. These people were often preoccupied with adapting to specific changes as direct result of their cancer and its treatment, rather than to improve their overall health.

Cause at the minute… the worst thing is the life change I get with the stoma bag. It [stoma] wasn’t really covered on in the Renewed, is it?...the stomach bag is my biggest bugbear…it’s just the stoma bag now is the thing that I’ve got to get over. *(Participant 21, low-user, male, colon cancer, Renewed arm)*

For a few patients, the COVID-19 pandemic was a barrier to motivation to use Renewed. This was because the pandemic introduced new concerns that took priority over cancer and related health behaviour change.

I think because of COVID the cancer has kind of taken a step back, it’s not been the priority or the focus as much as what it was. I work full-time, I’m a key worker so I have to do that. So my focus wasn’t on my cancer. *(Participant 25, low-user, female, breast cancer, Renewed arm)*

The COVID-19 pandemic was also a barrier to performing behaviour changes due to lockdown restrictions. This included physical limitations to going outside to exercise because of having to shield, or being physically limited in their ability to carry about behaviour changes due to contracting COVID-19.

When I had the COVID, when it first manifested itself, I was really very, very poorly. I couldn’t even lift the phone up, never mind look at a computer. *(Participant 33, high-user, male, colon cancer, Renewed with support arm)*

A minority of patients experienced technical issues such as navigation problems and error pages. This was reported when participants only had an iPad, with which Renewed was incompatible, or when certain technical bugs blocked participants’ access to components of Renewed. This only seemed to become a barrier to using Renewed in cases where the issue persisted to the extent that the individual could not effectively use the programme. For example, POWeR was a large stand-alone programme and could not be fully integrated into Renewed. This meant in order to access the POWeR intervention, users would be taken outside of the Renewed intervention, and in a few cases, participants experienced issues getting back into Renewed.

I did find most of the navigation was really good but I did find sometimes that when you went to an external site, like the POWeR, it was quite difficult to get back because there’s the button that says ‘take me back to Renewed’ I was hoping it’d take me back to the login page of Renewed but it didn’t. It took me back to the page I’d just visited which was the POWeR website. *(Participant 15, high-user, female, breast cancer, Renewed arm)*

Patients were warned that accessing POWeR would take them outside of Renewed, and very few patients expressed frustration in switching between Renewed and POWeR.

#### Personal touch and added value of human support

The majority of patients expressed a desire for some form of human support following their adjustment from finishing cancer treatment, whether from Renewed or elsewhere. In a few cases, it was expressed that there was a lack of understanding from others of how such support would be useful.

I’ve spoken to lots of people who do find that when they’re in remission after cancer, it’s almost as if everybody thinks, ‘Oh, that’s it’, you know, ‘You’re cured, you don’t need help anymore.’ but I do, I know a lot of people who do feel that people are not interested in how they’re getting on and whether they’re doing very well. *(Participant 11, high-user, female, colon cancer, Renewed with support arm)*

Many patients in the *Renewed with support* arm appreciated that Supporter sessions were available, believing it would provide an extra level of support and be beneficial for their rehabilitation and recovery.

It’s all very well doing something online, but if you’ve not got any support from anywhere else, I think it could be quite easy to go, “Oh yes, well I know this, I know this, and fine, I know what you’re getting at. But it’s just about having that personal touch, I think. *(Participant 18, low-user, male, prostate cancer, Renewed with support arm)*

A few patients in the *Renewed arm* (without access to additional support) expressed a desire for healthcare professional support offered alongside Renewed. They believed this would have provided extra support and made Renewed more personal.

You can’t pick up the phone and then talk to somebody about a specific problem…So I suppose that is where I fall down a bit with it…the ability perhaps to email somebody to discuss, might be something that ought to be considered added on. *(Participant 12, high-user, male, colon cancer, Renewed arm).*

Indeed, a few patients who expressed satisfaction with their Supporter, often appreciated that they were able to offer tailored advice and provide extra resources.

[I liked] the fact that she [Supporter] came up with some ideas. ‘Cause, as I say, she listened to me. But she came up with ideas in as much as things that she did, that I could implement. Which was to do with the, apart from walking for the papers, rather than getting all the fruit and veg when you do a main shop, getting it in-between time, and walking to the shop to get it. *(Participant 18, low-user, male, prostate cancer, Renewed with support arm).*

However, the perceived value and perceived need for support appeared dependent on existing social support. For the majority of participants who did not access support, this often seemed to be because they reported strong existing social support outside of Renewed, such as other medical professionals (i.e. cancer nurses), community support groups, charities or family and friends. Consequently, they often did not feel the need for extra support from Renewed.

I’m very lucky, I’ve got an excellent key worker at the hospital, yeah, and she’s been brilliant. So, she’s the one I’ve tended to go to. *(Participant 11, high-user, female, colon cancer, Renewed with support arm)*

There did appear to be some differences in the reported availability of existing social support in patients’ networks dependent on cancer type. Prostate cancer survivors generally reported less pre-existing support outside of Renewed compared to breast and colon cancer survivors.

A few prostate cancer survivors reported a lack of availability of support for managing the consequences of cancer and its treatment.

My absolutely perfect world would be to sit in a room with an oncologist, a cardiologist and some back specialist and for me just to talk to them for half an hour and say, “Look, these are all the things I want to do to feel better… And I feel like, okay, well that’s it. And there’s no place else to go. Which is quite frustrating. *(Participant 31, low-user, male, prostate cancer, Renewed arm)*

Prostate cancer survivors in the *Renewed arm* sometimes expressed that whilst being in the study provided a greater sense of social support, they would have preferred additional human support.

I feel that it [Renewed] can make you feel that you’re not completely on your own…it’s just having somewhere where some people who may be having this they don’t have any contact with other people…But also from that, I feel that it could be improved if somebody in the background within Renewed maybe should be contacting them [those using Renewed], maybe a health professional, because a lot of the time I find that I can go to a, my GP or whatever and I can write all my concerns or my questions down, but sometimes there’s no time to actually talk to them about problems. *(Participant 24, low-user, male, prostate cancer, Renewed arm)*

A few patients, across all cancer types, who started support sessions did not continue after their initial session because they were dissatisfied with their support. This was explored through deviant case analysis, finding that one participant disengaged from receiving support because he felt that the Supporter could not relate to, or understand his issues sufficiently to provide support. In this case, the patient spoke about sexual issues he was experiencing because of having had prostate cancer. The Supporter could not provide the support this patient needed and suggested that he speak to his GP or secondary care.

I think he found it difficult to relate to somebody of my age, especially with some of the problems with the type of cancer that I’ve got a lot of them things that revolved around the sexual side of my life. And I don’t think, he couldn’t cope with it. So, I got frustrated with that, and you know, there’s not a lot of point talking to him, ‘cause he actually doesn’t really understand what the problems are. *(Participant 6, high-user, male, prostate cancer, Renewed with support arm).*

Other reasons patients did not continue support included that they could not see any additional benefit. For example, one patient, who was having technical issues accessing POWeR, raised this issue with her Supporter and was told they would contact the study team to help resolve this issue for her, but she did not hear back from Supporter or Renewed study team. This experience made the patient feel dismissed as their Supporter was not able to follow through with the issues discussed, nor provide any encouragement or guidance.

I did say to him [Supporter] about the problems that I was having [accessing into POWeR], and he said he’d email somebody [from the Renewed study team], but I haven’t heard from anybody…I thought he might ask different questions about what would be helpful, or anything like that. But he didn’t. *(Participant 4, high-user, female, breast cancer, Renewed with support arm)*

## Discussion

This process study conducted qualitative interviews with cancer survivors who used Renewed to understand how and why they used Renewed as they did to allow a greater understanding of the Renewed RCT findings. These process study findings are discussed and triangulated with the RCT findings below.

A key aim of this study was to understand why some groups, like those with prostate cancer, might have benefitted more than those with breast and colorectal cancer in the Renewed RCT. Our findings suggest that the varying effectiveness of Renewed across cancer types may be at least in part due to differences in perceptions about the availability of, and perceived need for support in these individual’s lives. These perceptions of the availability of support outside of Renewed also seemed to relate to the extent to which people engaged with the intervention. Prostate cancer survivors spoke less about having social support outside of Renewed compared to participants with other cancers, who often expressed having adequate support elsewhere and so not needing Renewed as much. Those with prostate cancer also often reported having other health-related problems for which they expressed a lack of access to support. Previous research has similarly suggested that prostate cancer survivors generally feel under-supported [30], and being male has been associated with lower perceived social support across various cancers [31]. Furthermore, engagement with social networks can increase engagement in self-management among cancer survivors [32]. These findings suggest that the effect of Renewed in improving QoL compared to the control group among prostate cancer survivors may have been driven through Renewed providing the additional social support that this group felt they lacked outside of the intervention. In contrast, those with breast and colon cancers generally seemed to feel sufficiently supported already. In this study, prostate cancer survivors expressed a particular desire for professional advice and support, especially surrounding sexual health. Previous studies have suggested that prostate cancer survivors can find support though web-based interventions acceptable [33], as they may consider group support embarrassing and fear stigma of being vulnerable and emotional [34]. However, the human element offered by group support or peer support is valued for the informational and emotional exchange [35]. Therefore, an intervention like Renewed may be particularly acceptable to this group as it offers the privacy of an online interventions whilst providing the emotional and informational support through the option of HCP support.

Another key aim of this study was to explore any perceived changes in participants’ QoL and how these were experienced in relation to engagement with Renewed. This study was able to provide some understanding of the relationship between usage of Renewed and behaviour change, in as much that some participants reported not needing to access Renewed much before implementing behaviour changes, whilst others appeared to need to access Renewed more frequently before being able to implement behaviour changes. Many patients stopped using Renewed for various reasons whilst implying engagement with wider intervention goals, such as feeling as though they had received enough information to begin behaviour change or feeling sufficiently supported. For those who only used Renewed a little, using just the Core Content may be sufficient engagement with Renewed [11] for these individuals to provoke changes in behaviour. Those who did not use Renewed beyond the Core Content may not have perceived a need for more detailed and tailored support. This can potentially be understood through the concept of effective engagement [11], which recognises that the extent to which an individual actively uses an online intervention is not necessarily a direct reflection of their performance of behaviour changes recommended by that intervention. Some individuals may need to use the intervention less, whilst some may need to use it more before they are able to perform behaviours. This is in line with literature which suggests that users disengage from digital interventions when they obtain positive results, making further engagement redundant [36]. If an intervention like Renewed were to be adopted in primary care, it might provide suitable support for those with less need for resource-intensive support.

Another key aim of the study was to understand what factors may have served as potential barriers and facilitators to cancer survivors’ using Renewed and performing the recommended behaviours. One barrier identified was that many patients who disengaged early from Renewed did so due to an apparent lack of perceived need to use an intervention like Renewed. These patients expressed having access to sufficient support outside of Renewed, having finished treatment a whilst ago and thus not being focussed on their cancer symptoms anymore, or only participating for altruistic reasons connected with research participation, rather than because they wanted to make changes to improve their quality of life. Considering this finding in relation to the Health Belief Model [37] might offer greater understanding of patients’ decisions about whether or not to use Renewed. The Health Belief Model suggests that health-related behaviour change depends on several factors: perceived susceptibility, perceived severity, perceived benefits, perceived barriers, cues to action and self-efficacy. Especially relevant to these findings is the concept of perceived benefits. Perceived benefits refer to the belief in the efficacy of the recommended health behaviour in reducing the risk or seriousness of the condition—in this context, beliefs about the extent to which engaging with Renewed and following its recommendations were likely to improve their quality of life. This may suggest that those individuals who discussed having other resources to support them may have believed that those resources were sufficient or more efficacious in helping them manage their side effects, and so didn’t see the additional benefit of engaging with Renewed. It may also suggest that those who finished treatment a while ago or joined the study for altruistic reasons may not have perceived a benefit of using an intervention like Renewed because they had already built connections and knew how to manage their side effects, so an intervention like Renewed was not perceived as being able to further help them reduce the risk or seriousness of their side effects. If Renewed were provided outside of a research study, it is likely that these patients would not have taken it up, as they did not perceive a need for it. The Renewed intervention development work and the wider literature suggest that a subgroup of cancer survivors who desire to feel better after treatment want support to improve their quality of life [7]. However, others do not want to engage in behaviour change and are unlikely to become motivated to make changes, with or without access to resources like Renewed [6, 7, 38].

Another barrier which hindered some patients’ use of Renewed was a lack of perceived personal relevance. Many patients who did not access Renewed beyond the Core Content had already read widely about what they could do to help themselves and felt Renewed did not provide new information. Renewed was not considered relevant as it instructed them in things they already knew, instead of helping them learn new information. This is in line with findings from Kanera et al., (2016) which suggest perceived personal relevance is related to higher usage. Placing important and novel information early within an intervention may improve continued usage and exposure to behaviour change advice [39].

A few patients expressed not being able to engage in behaviour changes due to comorbidities. Despite this, the RCT results did not show less of an effect among those with comorbidities, which suggests that even if people are not able to follow all recommendations because of comorbidities, overall, this does not seem to prevent them from having some benefit from Renewed. Renewed was designed to be easy to use for those with comorbidities, so as not to be a barrier to engagement. However, as occurred in this study, mobility issues are particularly commonly reported as a barrier to physical activity among cancer survivors [40, 41]. Therefore, further work may be needed to develop content that can address concerns about engagement with physical activity whilst having mobility issues. It is noteworthy that COVID-19 often exacerbated these comorbid symptoms or introduced new illnesses within this sample. These illnesses due to COVID-19 reflect structural and psychological barriers to engagement. It may be that outside of the context of the pandemic patients may have had greater engagement.

## Strengths and limitations

This study provides useful insight into how an intervention like Renewed is experienced and may work to improve QoL among cancer survivors. There are a number of key strengths of this study. In line with guidance on conducting qualitative process studies alongside trials, data were collected and analysed iteratively [14]. This allowed issues underlying emerging themes to be explored further in later interviews. Furthermore, conducting interviews during the COVID-19 pandemic allowed unique experiences during that time to be identified, allowing data to consider contextual factors relating to users’ experience [14]. Consideration should be given to limitations; for example, it would have been useful to collect data on patients’ experiences over the duration of the study, rather than just during the first 3 months of being in the study. This might have allowed the identification of patterns over time and whether experiences changed [42]. However, after piloting interviews at different time points since participants had started Renewed, it was clear that data were richer (and participants memories clearer) when conducted within the first three months of using Renewed; therefore, the majority of the sample was interviewed at this time point. Additionally, it should be considered that there may have been other factors, other than level of Renewed usage, cancer type, age and gender, that may have influenced perceptions of Renewed and impact outcomes, experience and motivations to use the intervention. For example, research suggests those from minority ethnic groups and those from lower socio-economic backgrounds are less likely to engage in cancer research and digital interventions [43, 44]. However, those with a higher health literacy engage more with digital interventions have better outcomes [45]. However, despite attempts to do so, it was not possible to obtain as diverse a sample in socio-demographic characteristics. The experiences of using Renewed may have varied depending on these characteristics.

## Conclusion

This study has explored cancer survivors’ experiences using a digital intervention in primary care designed to improve QoL, and considered the findings alongside the results from the parallel RCT. These findings suggest that adding support alongside digital interventions may motivate engagement, particularly among those who lack this support outside of the intervention. Furthermore, these findings add to the literature regarding effective engagement with digital interventions, suggesting that even limited usage of online content may provide enough information to motivate behaviour change among those with less need for resource-intensive support. Novel information may need to be presented earlier in an intervention to motivate continued engagement with Renewed. This has implications for implementing Renewed and similar interventions into clinical practice as it suggests that with minor changes (e.g. addressing concerns about engagement with physical activity whilst having mobility issues), such an intervention may be able to provide support to many people with less need for intensive support and may be particularly helpful for those who lack support.

## Data Availability

The qualitative transcripts generated and/or analysed during the current study are not publicly available due to protecting participants’ anonymity.

## Declarations

### Disclaimer

The views expressed are those of the authors and not necessarily those of the NHS, the NIHR or the Department of Health and Social Care.
